# Ceramide-induced BOK promotes mitochondrial fission in preeclampsia

**DOI:** 10.1038/s41419-018-0360-0

**Published:** 2018-02-20

**Authors:** Jonathan Ausman, Joelcio Abbade, Leonardo Ermini, Abby Farrell, Andrea Tagliaferro, Martin Post, Isabella Caniggia

**Affiliations:** 1 Lunenfeld-Tanenbaum Research Institute, Sinai Health System, Toronto, ON M5T 1X5 Canada; 2 Institute of Medical Science, University of Toronto, Toronto, ON Canada; 3 Department of Obstetrics and Gynecology, Botucatu Medical School, UNESP – Sao Paulo State University, São Paulo, Brazil; 4 Department of Physiology, University of Toronto, Toronto, ON Canada; 5 Translational Medicine Program, Peter Gilgan Center for Research and Learning, The Hospital for Sick Children, Toronto, ON M5G 1X8 Canada; 6 Department of Obstetrics and Gynecology, University of Toronto, Toronto, ON Canada

## Abstract

Mitochondria are in a constant balance of fusing and dividing in response to cellular cues. Fusion creates healthy mitochondria, whereas fission results in removal of non-functional organelles. Changes in mitochondrial dynamics typify several human diseases. However, the contribution of mitochondrial dynamics to preeclampsia, a hypertensive disorder of pregnancy characterized by placental cell autophagy and death, remains unknown. Herein, we show that the mitochondrial dynamic balance in preeclamptic placentae is tilted toward fission (increased DRP1 expression/activation and decreased OPA1 expression). Increased phosphorylation of DRP1 (p-DRP1) in mitochondrial isolates from preeclamptic placentae and transmission electron microscopy corroborated augmented mitochondrial fragmentation in cytotrophoblast cells of PE placentae. Increased fission was accompanied by build-up of ceramides (CERs) in mitochondria from preeclamptic placentae relative to controls. Treatment of human choriocarcinoma JEG3 cells and primary isolated cytrophoblast cells with CER 16:0 enhanced mitochondrial fission. Loss- and gain-of-function experiments showed that Bcl-2 member BOK, whose expression is increased by CER, positively regulated p-DRP1/DRP1 and MFN2 expression, and localized mitochondrial fission events to the ER/MAM compartments. We also identified that the BH3 and transmembrane domains of BOK were vital for BOK regulation of fission. Moreover, we found that full-length PTEN-induced putative kinase 1 (PINK1) and Parkin, were elevated in mitochondria from PE placentae, implicating mitophagy as the process that degrades excess mitochondria fragments produced from CER/BOK-induced fission in preeclampsia. In summary, our study uncovered a novel CER/BOK-induced regulation of mitochondrial fission and its functional consequence for heightened trophoblast cell autophagy in preeclampsia.

## Introduction

Mitochondria are critical organelles that provide energy through oxidative phosphorylation^1^ and coordinate cell death via intrinsic apoptosis^2^. These ‘powerhouses’ are in a constant physiological balance of dividing and fusing; processes collectively known as mitochondrial dynamics. Mitochondrial fusion is a process that forms healthier and functional organelles from fragments with intact inner mitochondrial membrane (IMM) potentials^3^. Optic atrophy 1 (OPA1) and mitofusin 1 and 2 (MFN1/2) are key proteins involved in mitochondrial fusion that are responsible for bringing together the IMMs and outer mitochondrial membranes (OMMs) where they reside, respectively^4,5^. Alternatively, during fission, unhealthy, non-functional mitochondrial fragments, lacking transmembrane potentials, are discarded and targeted for degradation via a selective autophagic process termed mitophagy. The latter is dependent on the accumulation of phosphatase and tensin homolog (PTEN)-induced kinase 1 (PINK1) in the OMM, which recruits the E3 ubiquitin ligase Parkin, leading to mitophagy^6^.

Central to mitochondrial fission is the dynamin-related protein 1 (DRP1), an 80 kDa GTPase^7^. The activation of DRP1 occurs as a result of a number of post-translational modification events, most importantly phosphorylation of DRP1 (p-DRP1) at specific serine residue 616 leads to its activation and recruitment to the OMM where it interacts with resident proteins such as mitochondrial fission factor (MFF). This is followed by p-DRP1 oligomerization^8^ and consequent hydrolysis of GTP by active DRP1 providing the mechanic–enzymatic force by which fission occurs^9^. Typically mitochondrial fission occurs in highly metabolic subcellular regions termed mitochondria-associated endoplasmic reticulum membranes (MAMs)^10^. Interestingly, in addition to its role in mitochondrial fusion, MFN2 also plays a role in fission as this protein tethers together the mitochondria and endoplasmic reticulum (ER) forming the MAM^11^.

In humans, excessive mitochondrial fission has been implicated in the pathogenesis of several diseases^12^. *Drp1*^*−/−*^ mice exhibit embryonic lethality due to deficiency in the formation of trophoblast giant cells and consequent placental dysfunction, underscoring the requirement of mitochondrial fission for proper placental and embryonic development^13^.

Preeclampsia (PE) is a serious disorder that complicates 5–8% of pregnancies worldwide and represents a significant cause of maternal and fetal morbidity and mortality^14,15^. PE is typically characterized by excessive trophoblast cell death, generating a syncytial debris that is aberrantly extruded into the maternal circulation where it exerts a generalized endothelial inflammatory response clinically manifesting as hypertension^16^. To date, the involvement of mitochondrial dynamics in PE remains elusive.

We have reported that excessive cell death and autophagy in PE are in part dependent on a build-up of ceramides (CERs), a group of bioactive sphingolipids^17^. The accumulation of CER in PE has been shown to increase the expression of Bcl-2-related ovarian killer (BOK), a pro-apoptotic Bcl-2 family member, leading to increased trophoblast autophagy and death^17,18^. The altered MCL-1/BOK balance toward pro-death BOK has been implicated in the pathogenesis of PE^19^, although, to date, this has not been evaluated in the context of mitochondrial fission.

Herein, we report increased expression of key regulators of mitochondrial fission in PE. Furthermore, we attributed CER accumulation as a regulator of increased mitochondrial fission, through a novel mechanism involving BOK. Finally, we localized mitochondrial fission events to the ER/MAM compartments and show that the degradation of mitochondrial fragments in PE is occurring by PINK1/Parkin-mediated mitophagy.

## Results

### Mitochondrial fission is increased in PE

**Fig. 1:**
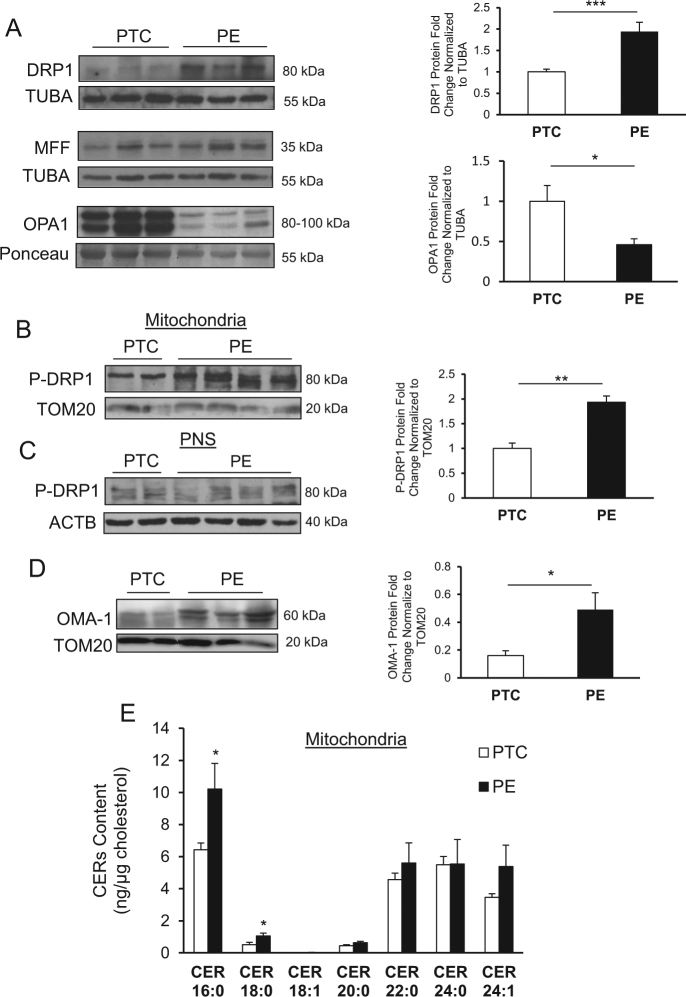
Changes in mitochondrial dynamics in preeclampsia associates with mitochondrial accumulation of ceramide. Representative western blots and associated densitometry of DRP1 (**a**, upper panel), MFF (**a**, middle panel), and OPA1 (**a**, lower panel) in PE vs. PTC. DRP1 WB and densitometry: PE, *n* = 30; PTC, *n* = 22; unpaired Student’s *t-*test ***P < 0.001. MFF WB: PE, *n* = 18; PTC, *n* = 9; unpaired Student’s *t*-test *P* = ns. OPA1 WB and densitometry: PE, *n* = 13; PTC, *n* = 10; unpaired Student’s *t-*test *P < 0.05). **b** Representative western blots and associated densitometry of p-DRP1 in mitochondria isolated from PE and PTC placentae (PE, *n* = 4; PTC, *n* = 4; unpaired Student’s* t*-test **P < 0.01). **c** p-DRP1 expression in the post-nuclear supernatant of PE vs. PTC placentae (PE, *n* = 4; PTC, *n* = 4). **d** OMA-1 expression in mitochondria isolated from PE and PTC placentae (PE, *n* = 8; PTC, *n* = 7; unpaired Student’s *t*-test *P < 0.05). **e** Ceramide levels normalized to cholesterol in mitochondria isolated from PE and PTC placentae as assessed by LC-MS/MS (PE, *n* = 4; PTC, *n* = 4; unpaired Student’s *t*-test *P < 0.05). All data are expressed as mean ± SEM (standard error of the mean)

We first examined the expression of DRP1, a key regulator of fission^12^, in placental tissues from PE and normotensive control pregnancies. Western blot (WB) analysis revealed significantly increased DRP1 levels in PE placentae relative to preterm controls (PTCs) (Fig. 1a, upper panel). Following its activation, DRP1 is recruited to MFF, a OMM-resident protein^20^. WB showed no changes in MFF levels in PE relative to PTC placentae (Fig. 1a, middle panel). We next examined the expression of OPA1, a key marker of fusion. WB analysis demonstrated a significant decrease in OPA1 expression in PE compared with PTC placentae (Fig. 1a, lower panel). Activation of DRP1 by phosphorylation at S616 residue is required for its mitochondrial recruitment where it triggers fission events^8^. Therefore, we isolated mitochondria from PE and PTC placentae and examined DRP1 activation using a specific antibody that recognizes phosphorylated DRP1 at S616 (p-DRP1). WB showed a significant increase of p-DRP1 in mitochondrial isolates (MIs) from PE relative to MI harvested from PTC placentae. Phosphorylated DRP1 levels were normalized to TOM20, a marker of the OMM (Fig. 1b). The post-nuclear supernatant (PNS), collected for comparison, showed no changes in p-DRP1 expression between the PNS of PTC and PE (Fig. 1c). Loss of mitochondrial membrane potential due to stress leads to the accumulation and activation of a peptidase termed OMA-1 that mediates OPA1 proteolytic cleavage thereby inhibiting mitochondrial fusion^21,22^. Hence, we examined OMA-1 content in MI using an antibody that recognizes the 60 kDa active form. WB revealed a significant increase in OMA-1 content in MI from PE compared with PTC (Fig. 1d), indicating that reduced OPA1 levels could be due to increased OMA-1 activity in the IMM. CER is a cell death inducer in PE leading to increased trophoblast autophagy^17^. Thus, we examined the CER content of MI from PE and PTC placentae using tandem mass spectrometry. A significant enrichment in CER 16:0 and CER 18:0 was observed in MI from PE relative to PTC placentae (Fig. 1e).

**Fig. 2:**
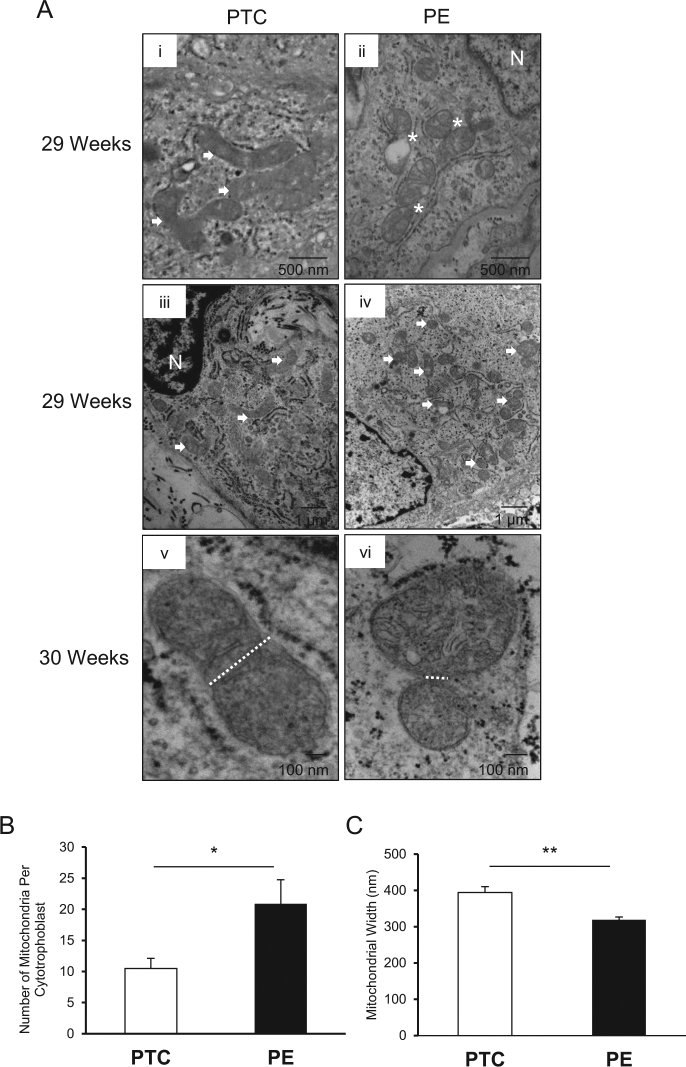
Preeclampsia is associated with mitochondrial fission morphology in cytotrophoblast cell. **a** Representative TEM images of cytotrophoblast cells from PE and PTC placentae from 29 to 30 weeks gestation. (i) Canonical mitochondrial morphology in PTC is identified by white arrows (scale bar: 500 nm), and (ii) mitochondrial fission events in PE are denoted by white stars (N, nucleus; scale bar: 500 nm). (iii/iv) Mitochondria in PE and PTC are indicated by white arrows (N, nucleus; scale bar: 1 µm). (v/vi) mitochondrial width is denoted by white dotted lines (scale bar: 100 nm). **b** Mitochondrial number per cell in PE vs. PTC (PE placentae, *n* = 8 (167 mitochondria); PTC placentae, *n* = 7 (63 mitochondria); unpaired Student’s *t*-test *P < 0.05), and **c** mitochondrial width in PE vs. PTC (PE, *n* = 8; PTC, *n* = 7 separate tissue samples; unpaired Student’s *t*-test **P < 0.01). Data are expressed as mean ± SEM (standard error of the mean)

Transmission electron microscopy (TEM) was employed for qualitative surveillance of mitochondrial morphology. Mitochondrial fission was identified by clear contact points between adjacent organelles and smaller globular mitochondrial fragments, in contrast to the elongated ovular morphology typical of healthy mitochondria, which exist in branching networks^23^. Augmented mitochondrial fission events were observed in cytotrophoblast cells from PE compared with PTC (Fig. 2a, i vs. ii). There was a twofold increase in the number of mitochondria per cytotrophoblast in PE compared with PTC (Fig. 2a, iii vs. iv; Fig. 2b). In addition, mitochondria from PE exhibited a significantly smaller mitochondrial width when compared with PTC (Fig. 2a, v vs. vi; Fig. 2c).

### CER increases DRP1 expression and activation

**Fig. 3:**
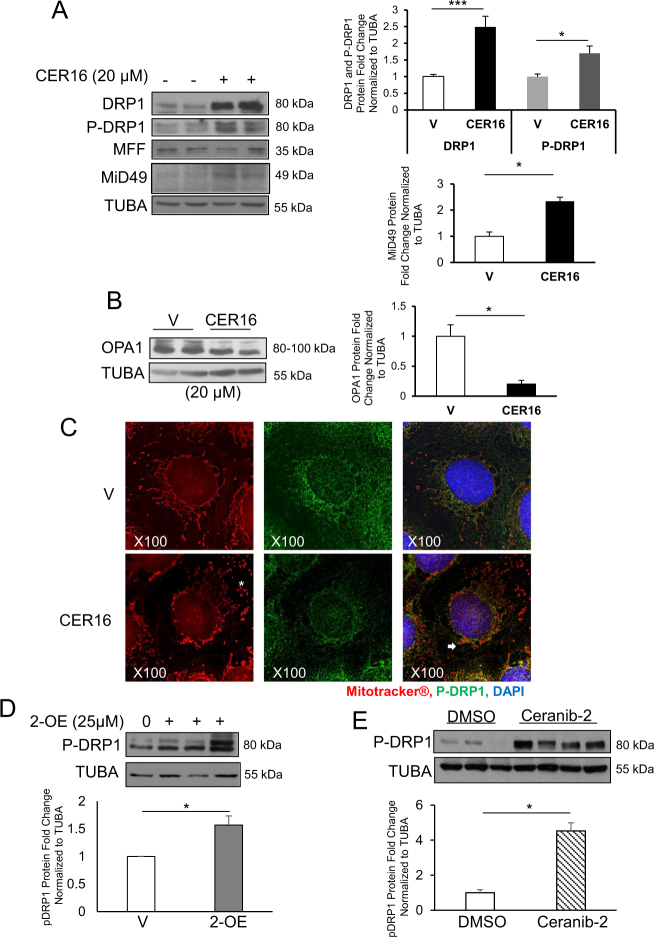
CER stimulates DRP1 expression and activation while reducing OPA1 levels in JEG3 cells. **a** Representative western blots of DRP1, p-DRP1, MFF, and MiD49 in JEG3 cells treated with CER 16:0 or EtOH vehicle (V) and associated densitometry (*n* = 10 separate experiments in duplicate; unpaired Student’s* t*-test *P < 0.05, ***P < 0.001). (**b**) Representative western blot of OPA1 in JEG3 cells treated with CER 16:0 or ETOH vehicle (*n* = 3 individual experiments in duplicate; unpaired Student’s* t*-test *P < 0.05). Data are expressed as mean ± SEM (standard error of the mean). **c** IF analysis of p-DRP1 in JEG3 cells treated with CER 16:0 or EtOH vehicle (V) and labeled with Mitotracker®. p-DRP1 (green), Mitotracker® (red), and nuclear DAPI (blue). **d** Representative western blot and densitometric analysis of p-DRP1 in JEG3 cells treated with 2-OE (25 µM) or control vehicle (*n* = 3 separate experiments; unpaired Student’s *t*-test *P < 0.05). **e** Immunoblotting of p-DRP1 and associated densitometry in placentae from CD1 mice injected with ceranib-2 (20 mg/kg), or DMSO vehicle (DMSO, *n* = 8; Ceranib-2, *n* = 9; *P < 0.05)

The presence of increased cytosolic, lysosomal,^17^ and mitochondrial CER in PE placentae prompted us to investigate the involvement of CER in mediating mitochondrial fission. DRP1 expression and phosphorylation were significantly increased in JEG3 cells following a 6-h treatment with 20 µM CER 16:0 relative to EtOH vehicle (Fig. 3a). CER 16:0 dosage and time were optimized in pilot experiments (Supplemental Fig. 1a). Similar to PE placentae, no changes in MFF expression levels were observed following CER 16:0 exposure (Fig. 3a). However, the content of another adaptor protein that recruits cytosolic DRP1 to the mitochondria, MiD49^24^, was increased in cells exposed to CER 16:0 (Fig. 3a). Interestingly, CER 16:0 markedly decreased OPA1 expression in JEG3 cells (Fig. 3b). Immunofluorescence (IF) analysis showed a striking redistribution and colocalization of p-DRP1 to the mitochondria (Mitotracker® Red) in JEG3 cells treated with CER 16:0 (Fig. 3c). Mitochondria from CER 16:0-treated JEG3 cells displayed a fragmented, globular morphology consistent with increased mitochondrial fission, when compared with cells treated with EtOH vehicle demonstrating mitochondria networks radiating from the nucleus (Fig. 3c). To further examine the contribution of CER in mitochondrial fission, we used 2-oleoylethanolamine (2-OE), an inhibitor of ASAH1 activity^25^ that increases autophagy in JEG3 cells^17^. Exposure of JEG3 cells to 25 µM 2-OE resulted in a significant increase in p-DRP1 levels (Fig. 3d). Similarly, administration of another ASAH1 inhibitor, Ceranib-2, to pregnant mice, which we showed to elevate CER content in the murine placentae similar to that found in PE placentae^17^, led to a significant increase in placental p-DRP1 (Fig. 3e).

**Fig. 4:**
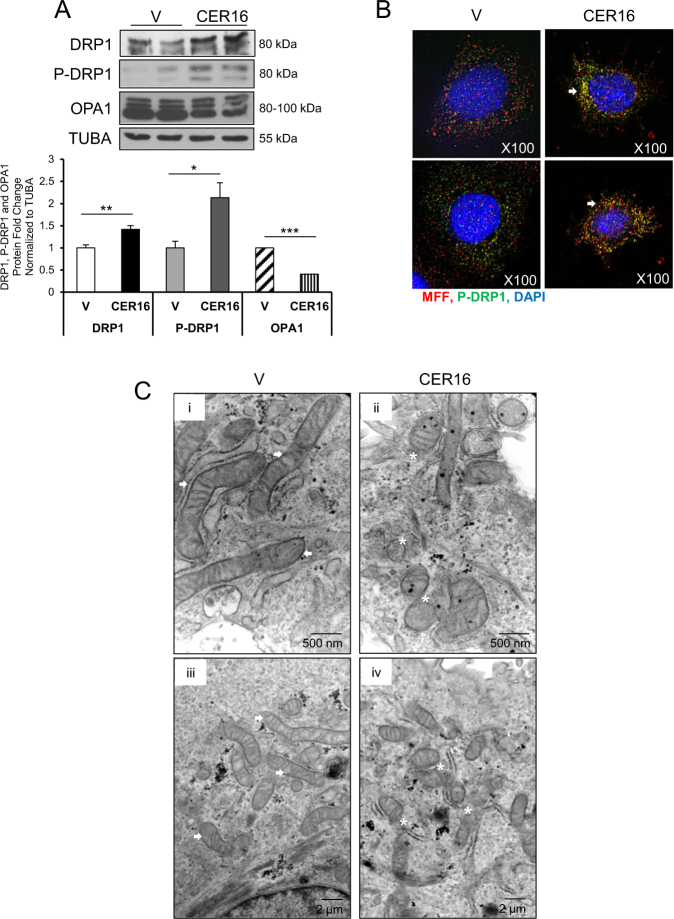
Ceramide induces mitochondrial fission in primary isolated trophoblast cells. **a** Representative western blots of DRP1, p-DRP1, and OPA1 and associated densitometry in primary isolated cytotrophoblast cells treated with CER 16:0 or EtOH vehicle (V) (*n* = 3 different primary cell isolations; *P < 0.05, **P < 0.01, ***P < 0.001; Data are expressed as mean ± SEM: standard error of the mean). **b** IF analysis of p-DRP1 (green) and MFF (red) in primary isolated cytotrophoblast cells following exposure to CER 16:0 or EtOH vehicle (V). Nuclei were stained with DAPI (blue). **c** Representative TEM of primary isolated cytotrophoblast cells from term placentae treated with CER 16:0 or ETOH vehicle (*n* = 5 different primary cell isolations). (i/iii) Mitochondrial morphology in vehicle-treated cells is identified by white arrows (scale bar: (i) 500 nm; (iii) 2 µm); (ii/iv) mitochondrial fission events and fragments in CER 16:0-treated cells are identified by white stars (scale bar: ii) 500 nm; (iv) 2 µm)

Similar to JEG3 cells, CER 16:0 treatment of primary isolated cytotrophoblasts resulted in a significant increase in both p-DRP1 and DRP1 relative to ETOH vehicle (Fig. 4a, upper and middle panels), and this associated with a decrease in OPA1 levels (Fig. 4a, lower panel). IF analysis showed that following CER 16:0 treatment, phosphorylated DRP1 was recruited to MFF on the OMM of primary isolated trophoblasts (Fig. 4b). p-DRP1 association with MFF following CER 16:0 treatment was corroborated by a Pearsons’ correlation coefficient (PCC) of 0.50 for the two fluorphores. In addition, TEM analysis in sections from primary isolated trophoblasts treated with CER 16:0 established the presence of increased globular mitochondrial fragments and fission events, when compared with the network-like mitochondria observed in controls (Fig. 4c).

### CER augments BOK-induced DRP1 expression

**Fig. 5:**
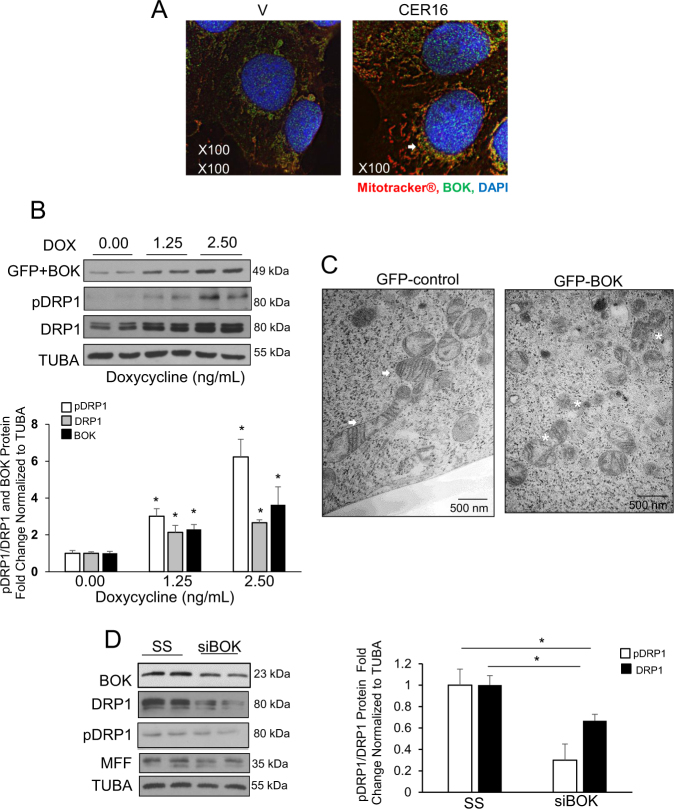
CER augments BOK-induced DRP1 expression leading to mitochondrial fragmentation. **a** IF analysis of BOK (green) in JEG3 cells treated with CER 16:0 or EtOH vehicle (V), and stained with Mitotracker® (red) and DAPI (blue). **b** Representative western blot and associated densitometry of BOK, p-DRP1, and DRP1 in HEK-293 cells stably transfected with GFP-BOK and induced with doxycycline (Dox 0, 1.25, or 2.5 ng/mL, *n* = 3 individual experiments carried out in duplicate; one-way ANOVA, Tukey’s post-test *P < 0.05, **P < 0.01). **c** Representative TEM images of HEK-293 cells stably transfected with GFP-BOK and induced with 2.5 ng/ml Dox (right panel) or treated with dH_2_O (control: left panel). Healthy mitochondrial morphology is indicated by white arrows, whereas mitochondrial fission events are depicted by white stars (scale bar: 500 nm; Dox 0 (vehicle control) and 2.5 ng/mL, *n* = 3 separate experiments). **d** Representative western blots and densitometric analysis of BOK, p-DRP1, DRP1, and MFF expression in HEK-293 cells following transient transfection with *BOK* siRNA or a scrambled sequence (SS); *n* = 3 separate experiments run in duplicate; unpaired Student’s *t*-test (*P < 0.05)

We have reported that CER-induced BOK is responsible for elevated trophoblast cell death and autophagy in PE^17^. CER 16:0 treatment triggered the expression and recruitment of BOK to the mitochondria in JEG3 cells (Fig. 5a). To examine the role of BOK in mitochondrial fission, we used an established human embryonic kidney 293 (HEK-293) Flp-In T-Rex cell system that allowed for the controlled expression of BOK upon doxycycline (Dox) stimulation^26^. Induction of BOK using Dox resulted in a significant increase in p-DRP1, DRP1, and BOK expression (Fig. 5b). Electron microscopy of GFP-BOK HEK-293-expressing cells revealed the presence of smaller, globular mitochondria, actively undergoing fission when compared with the larger mitochondria with well-defined cristae seen in the controls (Fig. 5c). Small interfering RNA (siRNA) knockdown of *BOK* in HEK-293 cells showed a significant decrease in p-DRP1, DRP1 levels compared with a scrambled control (Fig. 5d). Addition of CER 16:0 to cells following *BOK* siRNA treatment did not abrogated the knockdown effect on DRP1 (Supplemental Fig. 1b).

**Fig. 6:**
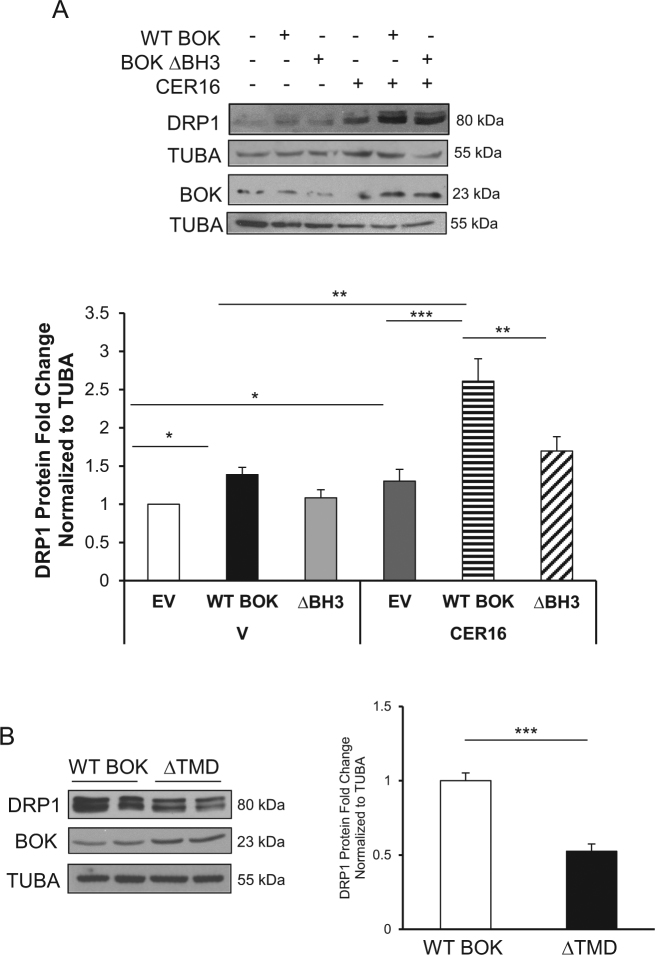
BH3 and TMD domains are responsible for BOK-induced mitochondrial fission. ) Representative western blots of DPR1 and BOK in HEK-293 cells transfected with plasmids containing empty vector (EV), WT BOK, and BOK-∆BH3, following exposure to CER 16:0 or EtOH vehicle (V). (**a**, lower panel) Densitometric analysis of DRP1 in HEK-293 cells transfected with EV, WT BOK, and BOK-∆BH3, following exposure to CER 16:0 or V, (*n* = 4 different experiments; one-way ANOVA, Tukey’s post-test *P < 0.05). **b** Representative western blot of DRP1 and BOK in HEK-293 cells stably transfected with inducible GFP-BOK-∆TMD with associated densitometry for DRP1 (Dox 0 (vehicle) and 2.5 ng/mL, *n* = 3 individual experiments in duplicate, ***P < 0.001)

To establish the relative contribution of the BH3 domain in mediating BOK’s effects on DRP1 expression, we transiently transfected HEK-293 cells with a plasmid-overexpressing BOK with a 17 base-pair deletion of the BH3 domain (∆BH3). In line with our inducible model, we found increased p-DRP1 and DRP1 levels in HEK-293 cells following transient overexpression of wild-type (WT) BOK relative to empty vector (EV) control (Fig. 6a and Supplemental Fig. 1c). In addition, CER 16:0 treatment further significantly augmented DRP1 expression in cells overexpressing WT BOK (Fig. 6a). Transient transfection of BOK, ∆BH3 resulted in significant less DRP1 expression compared with WT BOK (Fig. 6a). Beside the BH3 domain, BOK also contains a C-terminus transmembrane domain (TMD) that is critical for its mitochondrial translocation^27^ and depolarization. To ascertain its relevance on mitochondrial fission, we generated Dox-inducible HEK-293 cells that overexpress BOK with a deleted TMD. A significant decrease in DRP1 and p-DRP1 levels was observed in cells upon Dox induction that lacked the TMD relative to WT BOK controls (Fig. 6b and Supplemental Fig. 1C).

### CER induces BOK association with p-DRP1 at the MAMs

**Fig. 7:**
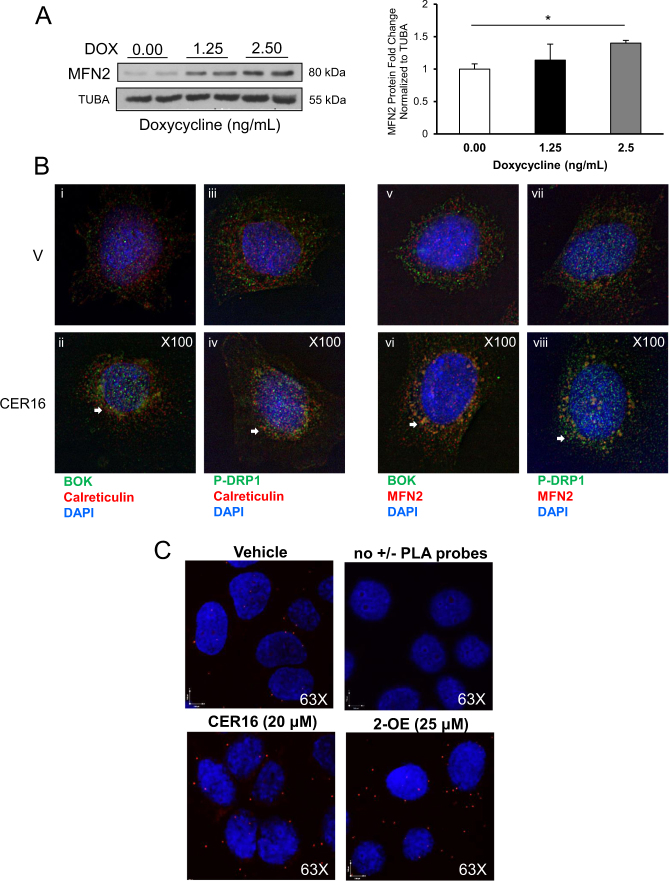
Ceramide triggers BOK association with p-DRP1 and MFN2 and VDAC1 to IP3R at the mitochondria-associated ER membranes. **a** Representative western blot and densitometry of MFN2 in HEK-293 cells stably transfected with GFP-BOK and induced with Dox (Dox: 0 (dH_2_O vehicle), 1.25 and 2.5 ng/mL, *n* = 4 separate experiments in duplicate; one-way ANOVA, Tukey’s post-test *P < 0.01). **b** Primary isolated cytotrophoblast cells treated with CER 16:0 or EtOH vehicle were stained for: (i/ii) BOK (green), calreticulin (red), and nuclear DAPI (blue) (*n* = 3 separate experiments); (iii/iv) p-DRP1 (green), calreticulin (red), and nuclear DAPI (blue) (*n* = 3 separate experiments); (v/vi) BOK (green), MFN2 (red), and nuclear DAPI (blue) (*n* = 3 separate experiments); and (vii/viii) p-DRP1 (green), MFN2 (red), and nuclear DAPI (blue) (*n* = 3 separate experiments). **c** Representative confocal images of in situ proximity ligation assay targeting VDAC1 and IP3R interactions in JEG3 cells exposed to CER 16:0 (20 μM) and 2-oleoylethanolamine (2-OE; 25 μM) for 6 h. Reactions without positive and negative PLA probes were used as negative controls

The MAMs are enriched in glycosphingolipids and represent the microenvironment that enables mitochondrial fission^28^. Tethering of the mitochondria to the ER is essential for MAM formation that requires MFN2^29^. BOK induction by Dox in HEK-293 cells stably transfected with GFP-BOK (Fig. 5b) resulted in increased MFN2 expression (Fig. 7a). Treatment of primary trophoblast cells with CER 16:0 resulted in a striking appearance of BOK and p-DRP1 in the ER/MAM compartments relative to control vehicle (Fig. 7b, i–iv) as assessed by calreticulin IF staining (MAM/ER marker) and association of both proteins with MFN2 (Fig. 7b, v–viii). Mean fluorescence intensity (MFI) analysis revealed an increase in p-DRP1 (1.78-fold), BOK (1.32-fold), and MFN2 (1.32-fold) in CER 16-treated cells relative to control vehicle. To convincingly demonstrate the importance of CER in promoting mitochondria-ER tethering, we employed in situ proximity ligation assay targeting voltage-dependent anion channel (VDAC1) and inositol 1,4,5-trisphosphate receptor (IP3R), two proteins found at the MAM interface^30^. Following a 6-h treatment with either CER 16:0 (20 μM) or 2-OE (25 μM) in JEG3 cells, we found a marked increase in the number of VDAC1/IP3R interactions points (Fig. 7c), indicating that excess CER increases mitochondria–ER tethering.

**Fig. 8:**
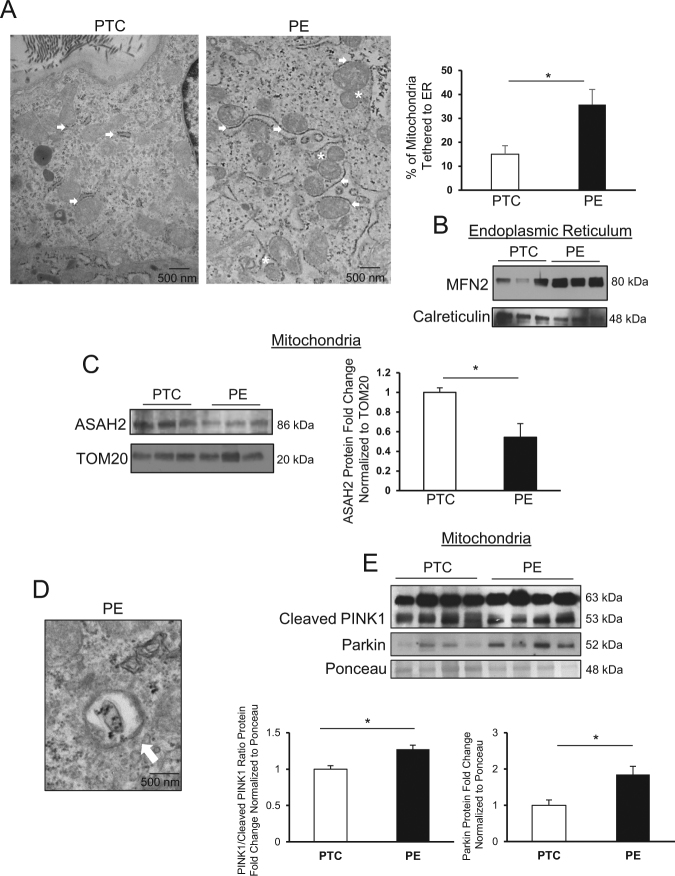
Mitophagy contributes to removal of excess mitochondrial fragments in preeclampsia. (**a**, left panel) Representative TEM image of a cytotrophoblast from PTC and PE placenta collected at 29 weeks. Mitochondrial proximity to ER (MAM) is indicated by white arrows, mitochondrial fission events are depicted by white stars (scale bar: 500 nm; *n* = 8 separate PE placentae). (**a**, right panel) percentage of mitochondrial tethering to the ER in in PE vs. PTC (PE placentae, *n* = 8; PTC placentae, *n* = 7; unpaired Student’s* t*-test *P < 0.05). **b** Representative western blots for MFN2 and calreticulin in ER isolated from PE and PTC placentae (PE and PTC, *n* = 3 separate samples). **c** WB and associated densitometry of ASAH2 (normalized to TOM20) in mitochondria from PTC and PE placentae. **d** Representative TEM depicting mitophagy (white arrow) in cytotrophoblast cell from PE placenta (scale bar: 500 nm, *n* = 8 separate PE placentae). **e** Western blot and associated densitometry of PINK1 and Parkin in PE vs. PTC mitochondrial isolates. Densitometry for PINK1 blot was used to calculate the ratio of full-length PINK_63kDa_ to cleaved PINK1_53kDa_ (PE and PTC, *n* = 4 separate placentae, *P < 0.05)

TEM analysis of PE placentae showed a significant increase in the presence of mitochondrial fission events in close proximity to the ER compared with PTC (Fig. 8a). In line with our observations of MFN2 accumulation in the ER of primary cells following CER 16:0 exposure, WB revealed increased MFN2 content in ER of PE placentae relative to PTC (Fig. 8b). No significant changes in different CER species were detected in MAM isolated from PE and PTC placentae (Supplemental Fig. 2). Our data indicates that CER accumulation in PE that is responsible for mitochondrial fission pertains to the mitochondria (Fig. 1e) and not to the ER/MAM. We next examined whether the increase in mitochondrial CER was due to changes in one of its regulatory enzymes, neutral ceramidase (ASAH2). Immunoblotting revealed decreased levels of ASAH2 (Fig. 8c) in mitochondria from PE placentae relative to PTC, suggesting that CER breakdown via ASAH2 is decreased in PE mitochondria leading to CER accumulation.

Mitophagy is a selective autophagic process that degrades non-functional mitochondrial fragments produced by fission^31^. TEM analysis of PE placentae identified mitophagy in cytotrophoblast cells (Fig. 8d). PINK1 is 63 kDa mitochondrial protein that is cleaved by PARL to an inactive 53 kDa isoform in the IMM; however, in damaged mitochondrial fragments, PINK1 cleavage is inhibited, and its 63 kDa isoform accumulates on the OMM where it phosphorylates cytoplasmic Parkin and ubiquitin resulting in the recruitment of the mitophagic machinery to carry out degradation^32^. Hence, we determined the PINK1_63kDa_/PINK1_53kDa_ ratio in MIs from PE and PTC placentae and found a significant increase in the pro-mitophagy 63kDa isoform relative to the non-active cleaved 53 kDa isoform in PE (Fig. 8e). Furthermore, immunoblotting showed increased mitochondrial Parkin levels in PE relative to PTC mitochondria (Fig. 8e).

## Discussion

In the present study, we demonstrate that mitochondrial fission occurs in the human placenta, and is augmented in PE. Furthermore, we show that CERs play a critical role in mitochondrial fission via a mechanism that involves BOK, a pro-apoptotic member of the Bcl-2 family. We identified the MAM as the microenvironment in which the interplay between BOK and key players of mitochondrial fission occurs, and that mitophagy is a cellular defense that removes excess mitochondrial fragments in PE.

Excessive DRP1-driven mitochondrial fission has been implicated in the pathogenesis of several human diseases, where accumulation of fragments with impaired mitochondria membrane potentials increases reactive oxygen species generation that overwhelms the inadequate antioxidant defenses^33^. Fission can participate in pathways leading to cell death, as seen in post myocardial infarction and in heritable juvenile Parkinsonism^12^, conditions associated with release of Ca^2+^ and^34^ loss of glutathione antioxidant defense^35^. PE placentae exhibit shallow trophoblast invasion and impaired transformation of maternal spiral arteries, which render the placenta vulnerable to hypoxia/oxidative stress^16^. Herein, we identified increased DRP1 expression, phosphorylation, and augmented mitochondrial fission events in placentae from pregnancies complicated by early onset PE, which we previously reported to have impaired oxygen sensing^36^ and elevated hypoxia-inducible factor 1-alpha (HIF1A) expression^37^. It should be noted that mitochondrial dynamics involve a balance between mitochondrial fission and fusion. In pulmonary arterial hypertension, increased HIF1A promotes DRP1-driven mitochondrial fission and fragmentation in human lung and pulmonary arterial smooth muscle cells, while decreasing MFN2 activity^38^. Loss of the fusion regulator OPA1 in HeLa cells has been found to alter mitochondrial membrane integrity and cristae remodeling, leading to increased mitochondrial fragmentation and apoptosis^39^. Conversely, transfection of HL-1 cells with mutant *Drp1*(*K38A*) abrogates mitochondrial fragmentation in a similar manner as MFN1 and MFN2 overexpression^40^. In support of the idea of a rheostat in mitochondrial dynamic events, herein we demonstrate that increased DRP1-dependent mitochondrial fission inversely correlates to fusion as identified by decreased OPA1 expression and augmented levels of active OMA-1 in PE placentae, and in trophoblast cells following CER 16:0 treatment. Our finding on impaired cell fusion in PE are in line with a study reporting downregulation of *MFN2* mRNA and impaired mitochondrial ATP production in PE placentae and in TEV-1 cells subjected to hypoxia^41^.

CERs are powerful inducers of intrinsic cell death in several systems^42^. We recently reported that specific CER species (eg., CER 16:0 and CER 18:0) are increased in PE placentae^17^. This increase in CER is dependent on the oxidative stress status of PE that impinge on CER regulatory enzymes, ultimately leading to increased trophoblast cell death rates^17^. Herein, we show that p-DRP1 is highly present in the mitochondria of PE placentae that are enriched in CER 16:0 and CER 18:0. Treatment of neonatal rat cardiomyocytes with synthetic CER 2:0 resulted in increased DRP1 expression and this was accompanied by a more spherical mitochondrial conformation favouring the initiation of apoptosis^43^. In line with this observation, we show that exposure of primary isolated cytotrophoblast cells—and JEG3 cells—to naturally occurring CER 16:0 increased DRP1 expression and phosphorylation, as well as p-DRP1 recruitment to the mitochondria, an event plausibly triggered by the presence of increased adaptor protein MiD49. Furthermore, we demonstrate that ASAH1 inhibition in JEG3 cells and in pregnant mice resulted in heightened p-DRP1 levels in cells and murine placentae, underscoring the importance of CER in the induction of mitochondrial fission in trophoblast cells during pregnancy.

Key to the formation of permeable channels at the mitochondria are the Bcl-2 family members, a group of proteins that act as either pro-apoptotic (BAK, BAX, and BOK) or pro-survival (Bcl-2, Bcl-XL, and Mcl-1) inducers^44,45^. The OMM produces CERs, which has been referred to as the ‘mitochondrial CER-rich macrodomain’ (MCRM), a platform by which BAX inserts, oligomerizes, and forms channels responsible for apoptosis in irradiated cells^46^. Mitochondrial fission is attenuated in primary neurons isolated from *Bak*-deficient mice brains, underscoring the importance of BAK as a regulator of fission^47^. Moreover, studies conducted in HeLa cells, demonstrated that DRP1 membrane association with the mitochondria is BAX/BAK dependent^48^, and BAX oligomerization is dependent on DRP1-induced membrane hemifusion, resulting in apoptosis^49^. Our present data highlight a novel and direct role for BOK on DRP1 expression and in fission events further underscoring the importance of pro-apoptotic Bcl-2 proteins in orchestrating mitochondrial dynamics. Notably, we have reported that accelerated trophoblast cell death rates, typical of PE, are due to high BOK levels^18,26,50^, and that CERs alter the BOK/MCL1 rheostat in favor of BOK leading to enhanced autophagy^17,18^.

Pro-apoptotic Bcl-2 family members can contain up to four Bcl-2 homology domains (BH1–4); however, the BH3 domain is crucial to apoptosis. Interestingly, its deletion in the Bcl-2/adenovirus E1B 19-kDa interacting protein 1 (BNIP1) results in diminished mitochondrial fission in HeLa cells^51^. Our current data on the abrogation of the BOK-induced effect on fission following transient overexpression of BOK-ΔBH3 in JEG3 cells further underscore the significance of this domain in regulating fission. Most Bcl-2 family members, including BOK, also contain a C-terminal α-helical TMD that functions to anchor the protein to the mitochondrial membranes aiding to its apoptotic function^52^. It has been reported that the TMD is critical for BOK recruitment to the ER and Golgi compartments^27^, and here we show that the TMD of BOK is also important for mitochondrial fragmentation likely by facilitating BOK recruitment to the ER/MAM compartments together with p-DRP1.

ER–mitochondria crosstalk is crucial for intracellular calcium signaling as it functions as a critical membrane contact site for lipid exchange and conversion^53^. MFN2 is the GTPase responsible for tethering the ER and mitochondria together^54^, although its most notable function is in mitochondrial fusion^5^. Of note, BOK has been shown to play a role in regulating the apoptotic response to ER stress^55^. Our present study demonstrates a significant increase in MFN2 protein following BOK induction. Hence, we propose that BOK increases MFN2 tethering between the ER and mitochondria to facilitate the process of mitochondrial fission. Notably, we found a marked increase in ER tethering to the mitochondria in PE and this associated with high MFN2 levels in the ER isolated from PE placentae. Emerging evidence suggests that CER produced in the ER is transported to the mitochondria via the MAM^44^. However, we did not find any CER changes between MAM isolated from PE and PTC placentae, indicating that the observed CER accumulation in the mitochondrial OMM is likely due to reduced breakdown of CERs. In support of the latter, we observed reduced mitochondrial levels of neutral ASAH2 (enzyme that hydrolysis CER to sphingosine) in PE placentae. We speculate that mitochondrial CER accumulation in PE results in more MCRM platforms in the OMM for BOK insertion thereby contributing to mitochondrial fission and trophoblast cell death.

Mitophagy is a highly specialized autophagic degradation pathway required to remove non-functional mitochondrial fragments^31^, and we have reported on the occurrence of mitophagy in PE placentae^18^. Mitophagy is classically dependent on PINK1 and Parkin^56^. In particular, mitochondrial fragments with impaired IMM potentials fail to import PINK1 to the IMM for cleavage, resulting in PINK1 accumulation to the OMM where it can recruit Parkin, which is responsible for OMM polyubiquitination required for mitophagy^57^. Herein we report increased levels of full-length PINK1 relative to its cleaved non-active isoform, and this is associated with increased Parkin levels in mitochondria isolated from PE placentae indicating that excess fragments are disposed by means of mitophagy. We propose that PINK1/Parkin regulated mitophagy is primed in PE likely as a defense against oxidative stress that typifies this pathology.

**Fig. 9:**
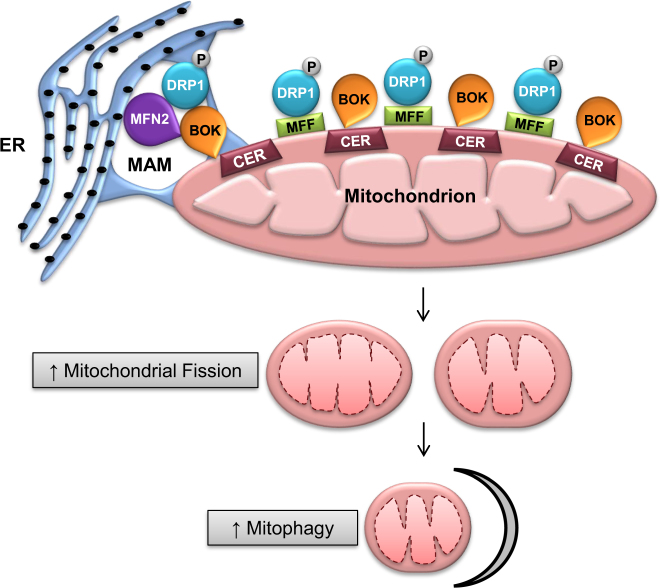
Putative model of the mechanisms underlying increased mitochondrial fission in PE. Putative model of the mechanisms underlying increased mitochondrial fission in PE. Elevated ceramide in mitochondria from PE pregnancies activates DRP1 and increases its recruitment to MFF at the OMM. p-DRP1 oligomerizes and completes the process of mitochondrial fission. Ceramide triggers the recruitment of BOK to the OMM, which; in turn, contributes to both augmented p-DRP1 expression and increases MAM tethering by inducing MFN2 expression. Mitochondrial fragments are degraded by means of PINK1/Parkin dependent mitophagy

Summarizing (see Fig. 9 for putative model), our data show that elevated CER in mitochondria from PE placentae, favors pro-apoptotic BOK recruitment to the OMM, and increased p-DRP1-dependent mitochondrial fission, resulting in elevated mitophagy. Thus, mitochondrial dynamic events favoring fission contribute to the exuberant cell death and autophagy characteristic of PE.

## Materials and methods

### Placental tissue collection

Informed consent was obtained from all clinical subjects, and placental collection was conducted in accordance with the ethical guidelines of the University of Toronto Faculty of Medicine and Mount Sinai Hospital by the Placenta BioBank, Mount Sinai Hospital, Toronto. All experiments are in agreement with the Helsinki Declaration of 1975, including its current 7th revision in 2013. The study was approved by the Mount Sinai Hospital Research Ethics Board (REB number: 11-0287-E). PE subjects (*n* = 33) were selected based upon the American College of Obstetrics and Gynecology (ACOG) criteria of maternal hypertension and proteinuria, or in the absence of proteinuria–thrombocytopenia, impaired liver function, pulmonary, renal, or cerebral disease^58^. Normotensive age-matched PTCs (*n* = 30) were selected based on the absence of placental disease with appropriate-for-gestational-age foetuses. Clinical parameters of PE and PTC subjects are listed in Table 1.

**Table 1:** Clinical parameters of the study population

Clinical parameters	Preterm controls (*n*=30)	Preeclampsia (*n*=33)
Gestational age at delivery (weeks)	29.7 ± 2.3	29.3 ± 3.0
Fetal weight (g)	1719 ± 282.2	1004 ± 372.0
Fetal weight (percentile)	0% ≤ 3rd	53% ≤ 3rd
Fetal sex	40% F, 60% M	33% F, 67% M
Systolic blood pressure (mmHg)	S: 114 ± 12.1	S: 170 ± 17.5
Diastolic blood pressure (mmHg)	D: 80 ± 8.3	D: 102 ± 11.5
Proteinuria (g/day)	Absent	3.6 ± 0.85
Mode of delivery	12.5% VD, 87.5% CS	54% VD, 46% CS
CS labor vs. CS non-labor	29% L, 71% NL	60% L, 40% NL

*F* female, *M* male, *S* systolic, *D* diastolic, *CS* cesareanc section, *VD* vaginal delivery, *L* labor, *NL* non-labor

### Transmission electron microscopy

PE (*n* = 8) and PTC (*n* = 7) placental tissue were collected and processed for TEM analysis immediately upon delivery. Primary isolated trophoblast cells from term placentae (*n* = 5) were treated with CER 16:0 (Avanti Polar Lipids) or EtOH vehicle, and HEK-293 cells stably induced with GFP-BOK were induced with Dox or dH_2_O as control (*n* = 3). Tissue and cell samples were fixed in 2% glutaraldehyde in 0.1 M cacodylate buffer (pH 7.3) for up to 24 h at 4 °C. The samples were processed by the Advanced Bioimaging Centre, Mount Sinai Hospital, Toronto. Placental tissue was processed into thin sections, and cells were embedded in coverslips containing Quetol resin (Electron Microscopy Scieneces, 20440), cut into 90 nm sections, picked up on copper grids and stained with uranyl acetate and lead citrate. Imaging was conducted on a FEI Technai 20 Transmission Electron Microscope.

TEM images of cytotrophoblast cells from PE (*n* = 8) and PTC (*n* = 7) placentae were obtained. For each placenta, three cytotrophoblast cells were identified, the number of mitochondria was counted, and an internal mean was generated. Mitochondrial width was measured using ImageJ® 1.49v software, where the minimum short-axis was recorded for each mitochondrion in at least three cytotrophoblast cells from all PE (*n* = 8) and PTC (*n* = 7) subjects. Statistical analysis was conducted as described below.

### Isolation of primary cytotrophoblast from term placentae

Whole, term placentae (*n* = 5), from normotensive, otherwise healthy women undergoing elective cesarean sections (C/S) for fetal malpresentation or previous C/S, were obtained within 10 min of delivery. Approximately 60 g of placental tissue was dissected, avoiding areas of calcification and large vasculature and was cut into smaller pieces. Primary cell isolation was carried out as previously described^59^, following a modification of Kliman methods^60^. Isolated cells were counted using Trypan blue and a hematocytometer, and cultured at a concentration of 1 × 10^7^ cells per 35 mm well, on coverslips for IF, or not for WB, in Dulbecco’s modified Eagle’s medium (DMEM) F:12 media (GIBCO-BRL, 11039-021) containing fetal bovine serum (FBS) and penicillin–streptomycin (Gibco®). Cells were cultured for 24 h at 8% pO_2_ (physiological oxygen tension for term placentae) and were subsequently treated with 20 µM synthetic CER 16:0 or EtOH vehicle for 6 h prior to collection for WB analysis, or fixation for IF in 4% paraformaldehyde solution.

### Cell culture and analysis

#### JEG3 human choriocarcinoma cells

JEG3 cells (ATCCHTB36^TM^) were cultured in six-well plates or coverslips in 20% O_2_ at 37°C in Eagle's Minimum Essential Medium (EMEM) media (ATCC, 30-2003) containing FBS and penicillin–streptomycin (Visent Inc). Once the cells attained 80% confluency, they were treated with either 20 µM CER 16:0 (Enzo Life Sciences, BML-SL115), 25 μM 2-OE (Invitrogen, 0383), or EtOH vehicle for 6 h, and either collected for protein analysis by WB, or fixed with 4% paraformaldehyde for IF.

#### HEK-293 cells

HEK-293 cells (ATCC®, CRL 1573^TM^) were cultured in high glucose DMEM media (Lunenfeld-Tanenbaum Research Institute, Toronto, Ontario) at 20% O_2_ at 37 °C to a confluency of 60–80%. Cells were used for transfection to silence (siRNA) and overexpress BOK, and to overexpress BOK ΔBH3 (described below). HEK-293 cells were stably transfected with GFP-hBOK using a Flp-In-T-Rex-293 cell line (ThermoFisher Scientific®) as previously described^26^. GFP-hBOK cells lines included WT and those with the following deletions: ΔBH3, ΔTMD. BOK-ΔBH3 plasmid was obtained by deletion of residues 65–82 corresponding to the BH3 domain of WT BOK. BOK-ΔTMD lacked the complete TMD domain. BOK WT and mutant expression was induced in the transfected cell lines by Dox at 1.5 or 2.5 ng/mL for 36 h.

### BOK transfection experiments

#### BOK silencing

HEK-293 were cultured as described above, and when a confluency of 60–80% was attained, cells were transfected with either 30 nM of Silencer® select siRNA targeted against the mRNA of BOK (Ambion, AM16708), or scrambled siRNA sequences as a control, using a jetPRIME® protocol (Polyplus Transfection®, 89129-922). Cells were cultured at 37°C and collected 24 h later for protein analysis by WB.

#### BOK/ΔBH3 overexpression experiments

HEK-293 cells were transfected with 2 µg/35 mm culture well of pcDNA BOK-L (WT BOK), pcDNA BOK-ΔBH3, and pcDNA3.1 (EV) (ThermoFisher Scientific®) using a jetPRIME® protocol. Protein was collected after incubation at 37°C for 24 h.

### Mitochondrial isolation

PE and PTC placentae were cut into smaller pieces, rinsed with isotonic saline (0.9% NaCl solution), and suspended in ice-cold buffer A (0.25 M sucrose, 0.001 M EDTA, 10 mM Tris-HEPES, pH 7.4). The tissue was subjected to two, 1-min homogenizations: one at low and the next at medium speed (Homogenizer: VWR®, 82027-184). The homogenate was centrifuged at 1300 *g* for 5 min at 4 °C and the supernatant (PNS) was further centrifuged at 12,000 *g* for 15 min at 4 °C, and yielded a MI pellet and post-mitochondrial supernatant (PMS). All three fractions (PNS, PMS, and MI) were validated using TOM20, a marker of the OMM, and β-actin (ACTB), a cytoskeletal protein marker. The MI was assessed biochemically by WB for p-DRP1 and a portion was used for CER analysis using liquid chromatography linked to tandem mass spectrometry (LC-MS/MS).

### MAM isolation

Subcellular fractionation and isolation of the MAM was carried out as previously described^61^. Briefly, the mitochondrial pellet of PE and PTC placentae isolated as described above was resuspended in 2 mL of EMEM media, and subsequently placed on a 30% Percoll gradient and centrifuged at 95,000 *g* for 30 min at 4 °C. The Percoll gradient was separated into the heavy fraction, containing the mitochondria, and the light fraction (LF) containing the MAMs. The LF fraction was centrifuged at 6300 *g* for 10 min at 4 °C, and the supernatant further centrifuged at 100,000 *g* for 1 h at 4 °C. The resulting pellet was the MAM isolate, which was validated by WB for absence of TOM20 and enrichment of calreticulin. DRP1 expression was evaluated by WB, and an aliquot was used for CER analysis by LC-MS/MS.

### CER measurements

MI and MAM isolates from PE and PTC placental tissues were processed for lipid extraction^62^ and CERs were measured by LC-MS/MS as previously described^17^. LC-MS/MS was performed at the Analytical Facility for Bioactive Molecules (The Hospital for Sick Children, Toronto) using an Agilent 1200 Series binary pump (Agilent Technologies Canada Inc.) linked to an API5500 triple-quadruple mass spectrometer (AB SCIEX).

### Mouse experiments

CD1 mice were purchased from Charles River (St. Constant, QC). Animal studies were conducted according to the criteria set up by the Canadian Council for Animal Care and approved by the Animal Care and Use Committee of the Hospital for Sick Children, Toronto, ON. Pregnant CD1 mice were intraperitoneally injected daily with Ceranib-2 (20 mg/kg; Cayman Chemical, 11092) commencing at E7.5 till E13.5. Ceranib-2 was dissolved in dimethyl sulfoxide (DMSO) and mice solely injected with DMSO were used as controls. At E13.5, placentae were snap frozen for biochemical analysis.

### WB analysis

WB analysis was conducted as previously described^63^. Briefly, PE and PTC snap-frozen tissue was pulverized in liquid nitrogen and homogenized in RIPA buffer (150 mM NaCl, 50 mM Tris, 1% NP-40, pH 7.5). The homogenate was centrifuged, and the supernatant transferred to a new tube for protein content analysis prior to WB analysis. Similarly, cultured cells were collected in 40 µL of RIPA buffer per well (of a six-well plate) and placed on ice for 1 h, centrifuged and the supernatant transferred to a new tube for protein content and WB analysis. The protein content of tissue and cell samples was assessed by Bradford protein assay (Bio-Rad®, 500-0006).

For WB, 30 µg of proteins from tissue and cell lysates were mixed with 8 µL of sample buffer (Tris 0.5% (pH 6.8), glycerol 20%, sodium dodecylsulfate (SDS) 10%, 2-β-mercaptoethanol, bromophenol blue 0.1%), and RIPA buffer to a total sample volume of 32 µL. Samples were subjected to sodium dodecyl sulfate–polyacrylamide gel electrophoresis and then transferred onto methanol-hydrated polyvinylidene fluoride membranes. The membranes were then blocked in 5% non-fat milk dissolved in tris-buffered saline (TBST) for 1 h, and left overnight in primary antibody at 4 °C. The next day, the membranes were washed three times for 15 min in TBST, and secondary antibody (horseradish peroxidase (HRP)-conjugated polyclonal antibody) was added for 1 h at room temperature. Blots were imaged using chemiluminescence ECL-plus reagent (PerkinElmer Inc., NEL103001EA) and X-ray film (GE Healthcare).

### IF analysis

Following experimental treatments, cells were fixed with 4% paraformaldehyde (Sigma®, F8775) for 15 min at 37 °C. Cells were permeabilized with 0.2% Triton X-100 for 5 min, rinsed with phosphate-buffered saline (PBS) and blocked with 5% normal horse serum (NHS) (Sigma®, H0146) for 1 h at room temperature. Primary antibodies were diluted in antibody diluent (0.4% sodium azide, 0.625% gelatin) and 5% NHS, and placed on cells for incubation overnight at 4 °C. For negative controls, the primary antibody was replaced with either nonimmune rabbit IgG (Santa Cruz Biotechnology, [sc-2027]) or goat IgG (sc-2028), corresponding to the primary antibodies being used. Following three PBS washes, HRP-conjugated secondary antibodies were diluted in antibody diluent and applied for 1 h at a concentration of 1:2000, after which three additional PBS washes was carried out. Cells were treated with 4’,6-diamino-2-phenylindole (DAPI) for 5 min to detect the nucleus, prior to fixation to 25 × 75 × 1 mm glass slides with Immuno-Mount™ (ThermoFisher Scientific®). IF images were obtained using a DeltaVision Deconvolution microscope (GE Healthcare). Live cell staining in JEG3 cells was conducted using 100 nM MitoTracker® red (ThermoFisher Scientific®), which was added for 5 min prior to fixation. IF quantification was performed using Volocity Software to determine either Mean Fluorescent Intensity or PCC.

### Proximity ligation assay

Duolink in situ Proximity Ligation Assay (Sigma Aldrich, USA) permits the detection of protein–protein interactions. JEG3 cells treated with and without CER 16:0 (20 μM) and 2-OE (25 μM) for 6 h, were cultured on eight-well chamber slides (LabTek, ThermoFisher, CA). Cells were then washed with PBS, fixed with cold 1:1 methanol and acetone for 3 min and permeabilized with 0.2% Triton X-100 for 5 min. Following a blocking step with Duolink blocking solution for 30 min at 37 °C, cells were incubated with VDAC1 and IP3R antibodies overnight at 4 °C. Hybridization of antibodies using plus and minus PLA probes raised against species of respective primary antibodies, ligation, and amplification reactions were performed according to the manufacturer's protocol. Slides were mounted with Duolink in situ mounting medium with DAPI (Sigma Aldrich, USA), and pictures were obtained using spinning disc confocal microscope with Volocity Imaging system.

### Antibodies

#### Primary antibodies

Commercially available primary antibodies were obtained for WB and IF analyses. Antibodies against DRP1 (sc-32898, rabbit [WB 1:1500]), MFF (T-14, sc-168593, goat [IF: 1:200, WB 1:1000]), OMA-1 (sc-515788, mouse monoclonal [WB 1:500]), MFN2 (H-68, sc-50331, rabbit [IF: 1:200, WB 1:1000]), BOK (H-151, sc-11424, rabbit [IF: 1:200, WB 1:1000]), TOM20 (FL-135, sc-11415, rabbit [WB 1:1000]), Neutral ceramidase (S-20, goat polyclonal; WB: 1:500), TUBA (αTubulin; P-16, sc-31779, goat [WB 1:2000]), and ACTB (β-actin; I-19, sc-1616, goat [WB 1:2000]) were purchased from Santa Cruz Biotechnology. Rabbit polyclonal anti-SMCR7 (Mid49) [WB 1:1000], mouse monoclonal anti-VDAC1 (ab14734) [PLA 1:200], and rabbit polyclonal anti-IP3R (ab5804) [PLA 1:200] were obtained from Abcam (Cambridge, UK). Antibodies against p-DRP1 (S616) (3455 S, rabbit [IF: 1:500, WB 1:1000]) were purchased from Cell Signalling Technology®. OPA1 (612607, mouse [WB 1:1000]) was purchased from BD Biosciences®; PINK1 (BC100-494, rabbit [WB 1:1000]) was purchased from Novus Biologicals®; and Parkin (AB9244, rabbit [WB 1:500]) was purchased from Millipore Sigma®.

#### Secondary antibodies

Secondary antibodies include goat anti-rabbit IgG-HRP (sc-2054 [WB: 1:2000]), donkey anti-goat IgG-HRP (sc-2056 [WB: 1:2000]), and goat anti-mouse IgG-HRP (sc-2005 [WB: 1:2000]) were purchased from Santa Cruz Biotechnology. For IF, Alexa Fluor® 488 donkey anti-rabbit IgG (A21206), Alexa Fluor® 594 donkey anti-rabbit IgG (A21207), Alexa Fluor® 488 donkey anti-goat IgG (A11055), Alexa Fluor® 594 donkey anti-goat IgG (A11058), and Alexa Fluor® 594 donkey anti-mouse IgG (A21203) were all purchased from ThermoFisher Scientific®.

### Densitometric and statistical analysis

WB densitometric analysis was conducted using ImageQuant® 5.0 software. Samples were normalized to either ACTB (β-Actin), TUBA (αTubulin), or Ponceau Stain. Statistical analysis was performed using GraphPad Prism 5 software, where comparison of two means utilized an unpaired Student's *t-*test, and comparison of multiple means used a one-way analysis of variance (ANOVA) with a Tukey post-test to compare two variables where applicable. Significance was denoted as *P < 0.05, **P < 0.01, and ***P < 0.001.

## Electronic supplementary material

Supplemental Figures
