# Supplementary material for: Ceramide-induced BOK promotes mitochondrial fission in preeclampsia
Source: Cell Death Dis. 2018 Feb 20;9(3):298. doi: 10.1038/s41419-018-0360-0 (PMC5833856; doi:10.1038/s41419-018-0360-0)
Supplement: Supplementary file 1 — Supplemental Figures [file 41419_2018_360_MOESM1_ESM.pdf]

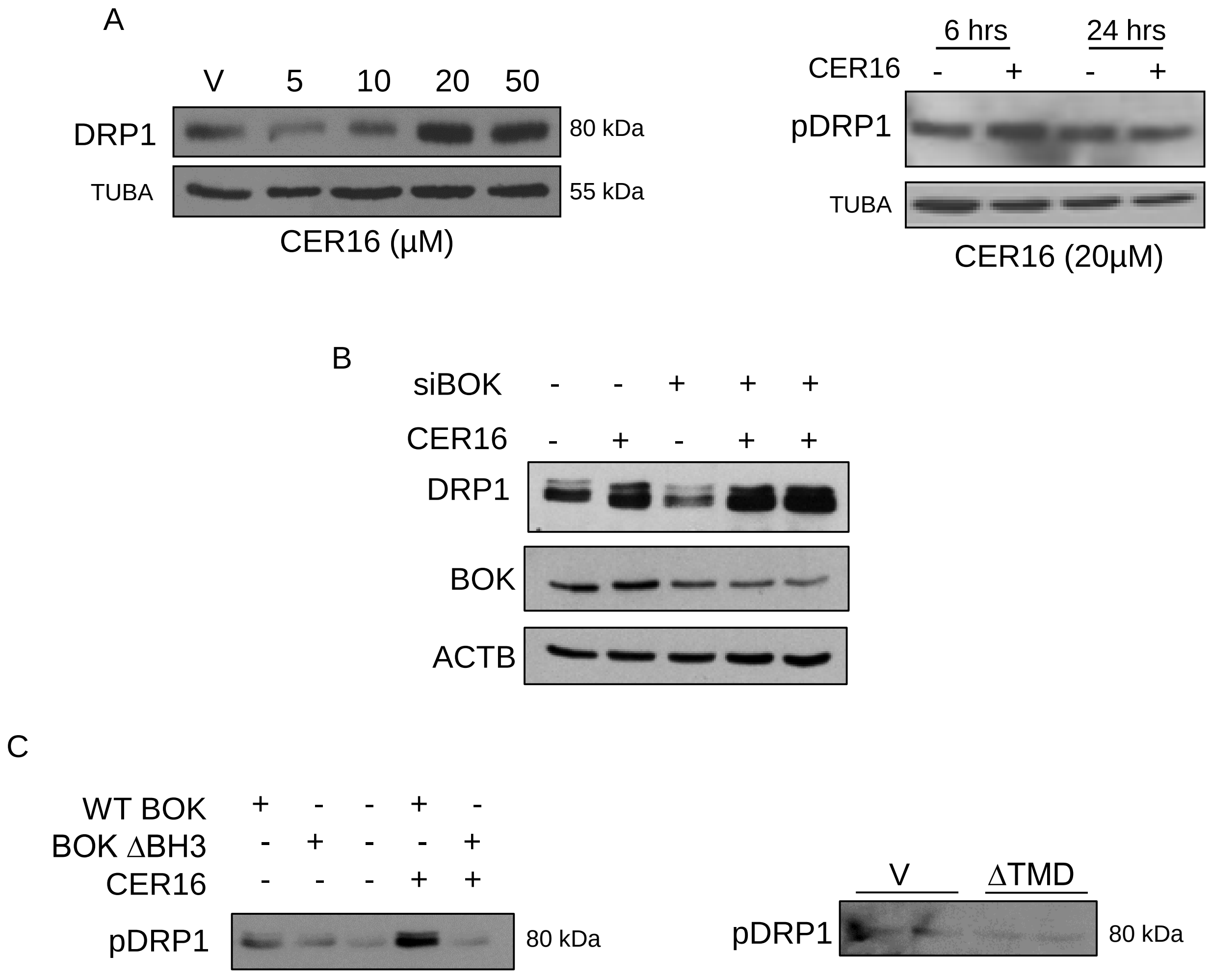

Supplemental Fig. 1 (**A**) Representative Western Blots of DRP1 in JEG3 cells exposed to different concentrations of CER16 (5-50 uM; left panel) and following CER16 treatment for 6 and 24 hours (right panel). (**B**) Immunoblotting of DRP1 and BOK in JEG3 cells following BOK silencing (siBOK) or treatment with control scrambled sequence, in the presence or absence of CER16 (20 uM). (**C**) Representative Western blot of pDPR1 in HEK-293 cells transfected with plasmids containing empty vector (-), WT BOK, and BOK-ΔBH3, following exposure to CER 16:0 (left panel). Representative western blot of pDRP1 in HEK-293 cells stably transfected with inducible GFP-BOK-ΔTMD (right panel).

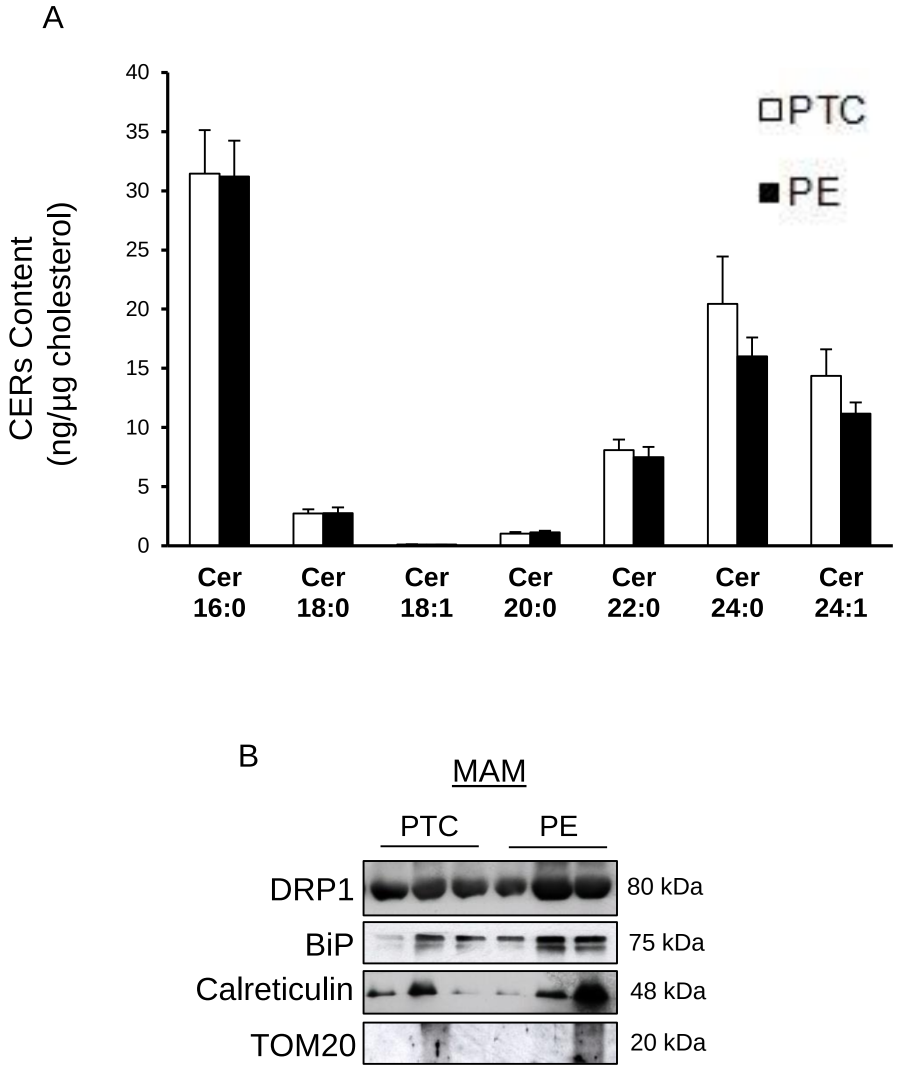

Supplemental Fig. 2. (**A**) LC-MS/MS analysis of ceramide normalized to cholesterol in MAMs isolated from PE and PTC placentae (PE and PTC, n=3 separate samples). (**B**) The purity of the isolated MAM fraction was established by the presence of DRP1, calreticulin, binding immunoglobulin protein (BiP), and absence of the mitochondrial marker TOM20 as assessed by Western Blotting (PE and PTC, n=3 separate samples). MAM: mitochondria associated membrane.
